# *In vitro* effect of mouthwash containing chlorhexidine and chlorine dioxide against halitosis pathogens

**DOI:** 10.2340/biid.v13.45222

**Published:** 2026-01-02

**Authors:** Thao Thi Phuong Tran, Thuy Anh Vu Pham

**Affiliations:** 1 Faculty of Odonto-Stomatology, Hong Bang International University, Ho Chi Minh City, Vietnam; 2 Faculty of Odonto-Stomatology, University of Health Sciences, Vietnam National University Ho Chi Minh City, Ho Chi Minh City, Vietnam

**Keywords:** Halitosis, chlorhexidine, chlorine dioxide, antimicrobial synergy, anaerobic bacteria

## Abstract

**Objective:**

This study evaluated the antibacterial efficacy of chlorhexidine-chlorine dioxide (CHX-CDO) mouthwash formulations against major halitosis-related pathogens: *Porphyromonas gingivalis (Pg), Aggregatibacter actinomycetemcomitans (Aa), Fusobacterium nucleatum (Fn), Prevotella intermedia (Pi)*, and *Eikenella corrodens (Ec)*.

**Materials and methods:**

Five bacterial strains were cultured anaerobically. Experimental mouthwashes with varying concentrations of CHX (0.01–0.2%) and CDO (0.05–0.1%), labeled solution A–J, were tested. Agar diffusion assays measured inhibition zones. MICs were determined by broth microdilution with 24 h anaerobic incubation and OD600 measurement. The time-kill test quantified CFUs from serial dilutions at time points (0, 1, 3, 6, 12, and 24 h).

**Results:**

All CHX-CDO mouthwash combinations inhibited bacterial growth, with A (0.05% CHX + 0.05% CDO), C (0.2% CHX + 0.05% CDO), F (0.2% CHX + 0.1% CDO), and J (CHX 0.02/CDO 0.1) showing the largest inhibition zones and G (CHX 0.01/CDO 0.05) and H (CHX 0.02/CDO 0.05) exhibiting the most favorable MIC values. Time-kill assays confirmed sustained bactericidal effects for low concentration-CHX/0.05% CDO formulations. Significant differences in antibacterial activity were observed among the formulations (*p* < 0.05).

**Conclusion:**

Low-dose CHX combined with 0.05% CDO mouthwash maintains antibacterial efficacy against halitosis-associated pathogens, suggesting a promising CHX dose reduction while preserving effectiveness.

KEY MESSAGES:

Low-dose CHX (0.01–0.02%) combined with CDO (0.05%) effectively inhibits halitosis-associated pathogens and may help reduce CHX-related side effects. This mouthwash represents a potent alternative to conventional 0.2% CHX mouthwashes, supporting the development of optimized dual-action mouth rinses for clinical use.

## Introduction

Halitosis, or bad breath, is a common condition that nearly half the population has experienced. The prevalence of the phenotype varies depending on the criteria used and the characteristics of the specific population being considered [1, 2]. Halitosis is the third most common reason for dental visits, following dental caries and periodontal disease, and it can adversely affect patients’ social interactions and psychological well-being [1]. In approximately 80–90% of cases, halitosis originates from the oral cavity, where specific anaerobic bacteria such as *Prevotella intermedia (Pi), Aggregatibacter actinomycetemcomitans (Aa), Porphyromonas gingivalis (Pg), Fusobacterium nucleatum (Fn),* and *Eikenella corrodens (Ec)* degrade sulfur-containing amino acids into volatile sulfur compounds (VSCs), including hydrogen sulfide (H_2_S), methyl mercaptan (CH_3_SH), and dimethyl sulfide (CH_3_SCH_3_) [3, 4]. While H_2_S and CH_3_SH are mainly associated with intra-oral malodor, CH_3_SCH_3_ is considered a marker of extra-oral or blood-borne halitosis, as it is not predominantly produced by oral microorganisms [5].

Maintaining good oral hygiene by combining mechanical brushing with chemical products is usually necessary for managing halitosis [4]. Regarding antimicrobial agents, chlorhexidine (CHX) remains the ‘gold standard’ for its long-lasting surface retention and ability to effectively eradicate a wide range of bacteria. However, according to the literature, long-term CHX use causes specific adverse side effects, including discoloration of the tongue and teeth, changed taste, and slowed wound healing [6]. On the other hand, chlorine dioxide (CDO) is a potent yet safe oxidizing agent that can rapidly lower VSC levels without causing significant adverse effects [7].

Although CHX and CDO are commonly used separately, few studies have examined their combined effect. CHX and CDO, when used appropriately, may neutralize VSCs and eradicate microorganisms while adhering to surfaces. This unique mixture may counteract the adverse effects of each component, depending on their concentrations. Recent research has shown that CHX and CDO can be combined without reaction to yield inert compounds or precipitates, consistent with the concept of a dual-action mouthwash [8, 9]. The combination of CHX-CDO has not yet been investigated for its susceptibility to the major halitosis-related anaerobic microorganisms, creating a significant gap in the literature. In this study, the antibacterial properties of CHX-CDO formulations at varying doses will be evaluated using agar diffusion, minimum inhibitory concentration (MIC), and time-kill assays.

## Materials and methods

### Materials preparations

The experimental mouthwashes were prepared by combining chlorhexidine digluconate (CHX) with CDO in varying proportions. Stock solutions of CHX 20% (Bajaj Healthcare, India) and CDO 5% (Shinwang, South Korea) were utilized to formulate test preparations.

Five oral bacterial strains linked to halitosis and periodontal disease were included: *Porphyromonas gingivalis (Pg), Aggregatibacter actinomycetemcomitans (Aa), and Fusobacterium nucleatum (Fn),* which were clinical isolates derived from the subgingival plaque of moderate to severe periodontitis patients and preserved by cryopreservation [10–12]. Subgingival plaque samples were collected using sterile paper points (MP201-S607, size 35, DiaDent, South Korea) and subsequently cultured anaerobically at 37°C for 3–5 days (80–90% N_2_, 5% H_2_, and 5–10% CO_2_) using GasPak^®^ anaerobic systems (Merck, Germany) and AnaeroPack^®^ sachets (Mitsubishi, Japan). Bacterial identification was first performed using MALDI-TOF mass spectrometry and then confirmed by PCR with species-specific primers. PCR amplification was conducted on an ABI GeneAmp^®^ system, and amplicons were separated by electrophoresis using a HyperLadder™ 100 bp molecular weight marker. The PCR products were subsequently analyzed using an ABI 3730XL Genetic Analyzer (Applied Biosystems, Malaysia).

*Eikenella corrodens (Ec)* (ATCC^®^ BAA-1152) and *Prevotella intermedia (Pi)* (ATCC^®^ 25611) were obtained from Microbiologics (USA). They were grown in Wilkin–Chalgren anaerobic agar (CM0619, Oxoid Ltd., Hants, United Kingdom) supplemented with 5% sheep blood (Nam Khoa Biotek Company Limited, Vietnam) at 37°C under anaerobic conditions (80- 90% N_2_, 5% H_2_, 5–10% CO_2_) for 72 h. For practical use, the bacterial suspension was diluted to 1 × 10^6^ or 1 × 10^5^ colony-forming units (CFU) mL^-1^. The culture medium was also prepared from Wilkins-Chalgren Anaerobe Broth (CM0643, Oxoid Ltd., Hants, United Kingdom) for the test.

### The agar diffusion assay

Ten solutions with different concentrations of CHX and CDO were prepared as described in Table 1. Positive controls consisted of CHX 0.2% and CDO 0.1%, whereas 0.9% NaCl served as the negative control.

**Table 1:** Composition of test mouthwash formulations.

ID	A	B	C	D	E	F	G	H	I	J
CHX (%)	0.05	0.1	0.2	0.05	0.1	0.2	0.01	0.02	0.01	0.02
CDO (%)	0.05	0.05	0.05	0.1	0.1	0.1	0.05	0.05	0.1	0.1

Plates were bacterially inoculated with sterile cotton swabs, and 6-mm wells were created in the agar. Each well was administered 100 µL of the test solution. The samples were incubated anaerobically at 37°C for 24 h. Inhibition zones were captured and analyzed utilizing ImageJ software (version 1.54p, National Institutes of Health, Maryland, United States). Each condition was evaluated in triplicate.

### Assessment of the MIC

Based on the agar diffusion assay results, the mouthwash formulations exhibiting measurable antimicrobial activity at lower concentrations were selected for MIC testing.

This assay was conducted utilizing the broth microdilution technique in 96-well plates. Triplicate two-fold serial dilutions of each mouthwash were performed. A standardized bacterial suspension (5 × 10⁵ CFU/mL) was added to each well (20 µL), along with 180 µL of diluted test solution. Wells containing only medium (bacteria-free) served as negative controls, while wells containing bacteria but no test solution served as positive controls. Plates were incubated anaerobically at 37°C for 24 h, and optical density was measured at 600 nm using a microplate reader (Microlab Plus, India). MIC was determined as the minimum concentration at which no growth was observed (OD₆₀₀ equivalent to that of the negative control), and MIC values were presented as serial two-fold dilutions.

### Time-kill assay

Time-kill assays were performed using the mouthwash formulation that exhibited the most promising antimicrobial activity in both the agar diffusion and MIC tests. The assay was conducted at the MIC for each bacterial strain.

A total volume of 2 mL, consisting of 800 µL of the appropriately diluted mouthwash (at MIC), 1,190 µL of culture medium, and 10 µL of bacterial suspension (10⁸ CFU/mL), was incubated anaerobically at 37°C. Samples were withdrawn at 0, 1, 3, 6, 12, and 24 h; serially diluted; and plated on blood agar to determine CFU. Control tubes without mouthwash were included. Bactericidal activity was expressed as the log₁₀ reduction in CFU over time. Each experiment was conducted in triplicate, yielding consistent and reproducible results.

### Statistical analysis

All data were analyzed using IBM SPSS Statistics version 30.0 (IBM Corp., Armonk, New York, United States). Normality was assessed using the Shapiro–Wilk test, and homogeneity of variance was evaluated with Levene’s test. Since Levene’s test confirmed the assumption of homogeneity (p > 0.05) and the results of both one-way analysis of variance (ANOVA) and Kruskal–Wallis tests were consistent, a parametric ANOVA followed by Tukey’s honestly significant difference (HSD) post hoc test was used to identify statistically significant differences among test solutions for each bacterial strain.

## Results

### Agar diffusion assay

**Figure 1:**
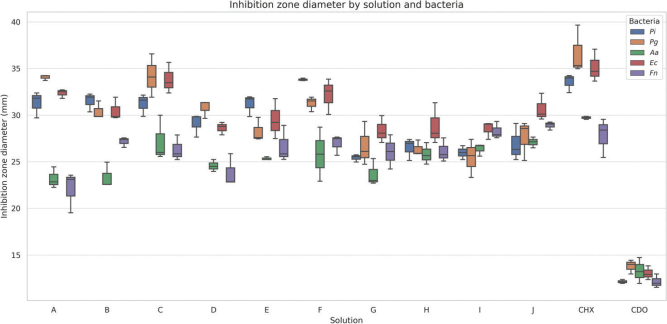
Boxplot of inhibition zone diameters (mm) for 12 mouthwash formulations (Solutions A–J, CHX, and CDO) tested against five oral malodor-associated bacteria (*Pi, Pg, Aa, Ec, and Fn*).

Figure 1 shows the inhibition zone diameters of solutions A–J against five halitosis-associated bacteria. Statistical analysis (ANOVA) confirmed significant differences in antibacterial activity among the formulations for all tested bacteria (*p* < 0.05), with the most potent effects observed against *Pg*, *Fn*, and *Pi* (*p* < 0.001).

Solutions A (CHX 0.05% + CDO 0.05%), C (CHX 0.2% + CDO 0.05%), F (CHX 0.2% + CDO 0.1%), and J (CHX 0.02% + CDO 0.1%) produced the largest inhibition zones (mean >30 mm), particularly against *Pg* and *Fn*. Solution J demonstrated the highest effect against *Pg* (about 39 mm), significantly greater than solutions B, E, and G (*p* < 0.005, Tukey HSD). It also showed consistent effects on *Pi* and *Fn*. Solutions G, H, and I showed moderate activity (28–33 mm), while solutions B (CHX 0.1% + CDO 0.05%) and E (CHX 0.1% + CDO 0.1%) had significantly smaller zones than most others (*p* < 0.01), particularly against *Pg, Pi,* and *Ec*.

The positive control (CHX 0.2%) showed significant and uniform inhibition across all strains, whereas CDO (0.1%) alone exhibited the weakest activity, especially against *Aa* and *Ec*.

### MIC assay

CHX 0.2% demonstrated the most potent antibacterial activity, with the lowest MIC values across all species (ranging from <1/512 to 1/128). Among the CHX–CDO combinations, solution H (CHX 0.02% + CDO 0.05%) showed the best overall MIC profile, exhibiting the lowest MIC against *Aa* (1/32) and comparable potency against *Pg*, *Ec,* and *Fn* (1/8–1/16). Solution G (CHX 0.01% + CDO 0.05%) was also highly effective, particularly against *Pg* (1/8). Solution I and J (with a higher CDO concentration of 0.1%) showed more potent inhibition toward Ec (1/32) but did not consistently outperform solutions G and H for the remaining species (Table 2).

**Table 2:** Minimum inhibitory concentration of test mouthwash formulations.

	*Aa*	*Pg*	*Ec*	*Pi*	*Fn*
Solution G	1/16	1/8	1/16	1/4	1/8
Solution H	1/32	1/8	1/16	1/8	1/8
Solution I	1/16	1/16	1/32	1/8	1/8
Solution J	1/16	1/16	1/32	1/4	1/8
CDO 0.1%	1/16	1/8	1/4	1/8	½
CHX 0.2%	<1/512	1/512	1/512	1/128	1/256

These results suggest that low-CHX formulations containing 0.05% CDO (G and H) maintain broad-spectrum antibacterial activity comparable to high CHX levels.

### Time-kill assay

**Figure 2:**
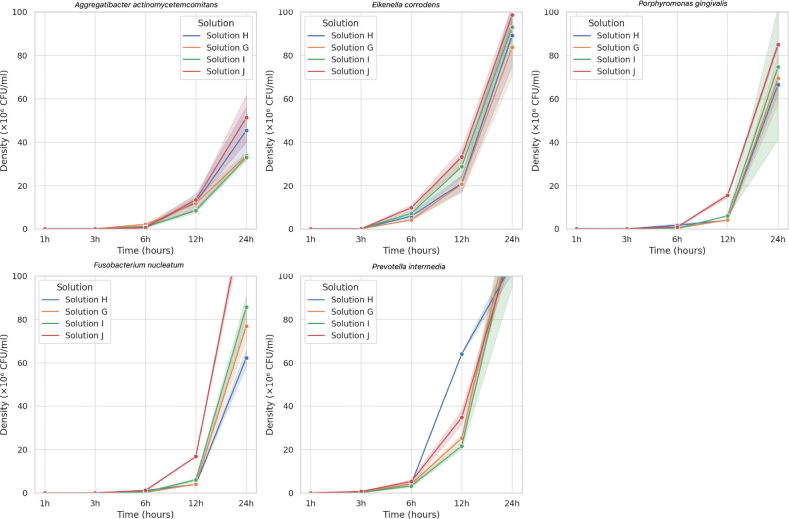
Growth curves of five halitosis-associated bacterial species treated with four mouthwash formulations, G-J, over 24 hours.

In the time-kill assay, solutions G and H (CHX 0.01–0.02% + CDO 0.05%) maintained the lowest CFU counts across the 24 h, particularly against *Pg* and *Ec*. Statistically significant differences in bacterial regrowth were observed among the four formulations at six h and 24 h (*p* < 0.01). Solutions I and J (containing higher CDO concentrations of 0.1%) exhibited partial regrowth after 6 h, especially in *Pi* and *Fn* (Figure 2).

## Discussion

This study investigated the antibacterial efficacy of mouthwash formulations containing chlorhexidine and CDO against five anaerobic bacterial species commonly associated with halitosis, namely, *Porphyromonas gingivalis* (*Pg), Aggregatibacter actinomycetemcomitans (Aa), Fusobacterium nucleatum (Fn), Prevotella intermedia (Pi),* and *Eikenella corrodens (Ec)*. The findings confirm that CHX at 0.2% exhibited the most potent antibacterial activity among all tested solutions, consistently producing the largest inhibition zones across all bacterial species. These results are consistent with earlier reports, demonstrating CHX’s potent antimicrobial effect through membrane disruption, cytoplasmic precipitation, and interference with intracellular protein function [13, 14].

Although CDO alone (0.1%) showed measurable antibacterial activity, it was consistently less effective than CHX 0.2% and most CHX-CDO combinations. This observation may be attributed to the high volatility and instability of CDO, particularly in open systems such as agar diffusion assays. Several studies have shown that CDO rapidly dissipates in air and aqueous environments, resulting in reduced diffusion and shorter contact times on agar surfaces [15, 16]. Additionally, the strict anaerobic, Gram-negative bacteria used in this study are generally more resistant to oxidative stress than facultative anaerobes or aerobes, further explaining the lower efficacy of CDO in this setting.

Interestingly, several dual-agent formulations with reduced CHX concentrations still demonstrated strong antibacterial effects. Among them, solution H (0.02% CHX + 0.05% CDO) presented the most favorable overall MIC profile, showing the lowest MIC for *Aa* (1/32) and comparable inhibition of *Pg*, *Pi*, *Ec*, and *Fn* (1/8–1/16). Solution G (0.01% CHX + 0.05% CDO) also performed effectively, particularly against *P. gingivalis* (1/8). In contrast, solutions containing a higher CDO level (0.1%; I and J) improved the susceptibility of *Ec* (1/32) but did not consistently outperform solutions G and H for other species. These findings indicate that an optimized CHX-CDO ratio, rather than simply increasing CDO concentration, is essential for maximizing antimicrobial effectiveness while reducing the CHX dose.

The potential antimicrobial effects of CHX and CDO are likely related to their complementary mechanisms of action. CHX is a cationic bisbiguanide that binds to bacterial cell walls, altering membrane permeability and, at high concentrations, causing cell lysis [13]. In contrast, CDO acts as a nonspecific oxidizing agent that reacts with sulfur-containing amino acids such as cysteine and methionine, disrupting enzymatic and structural protein functions within microbial cells [17]. When combined at optimized concentrations, these agents may exert additive or even synergistic effects. However, the efficacy of such combinations depends heavily on maintaining the chemical stability of both agents.

At higher concentrations, CDO becomes less stable in aqueous solutions, degrading into chlorite (ClO_2_⁻) and chlorate (ClO_3_⁻), both of which possess reduced antimicrobial activity. Moreover, excessive oxidative stress from high CDO levels may lead to nonspecific reactivity, reducing target specificity and potentially interfering with the stability or function of CHX in the [18–20]. This phenomenon could explain why formulations with 0.1% CDO, such as solutions I and J, did not consistently outperform those with 0.05% CDO.

The importance of optimizing both the concentration and ratio of CHX and CDO has been emphasized in previous studies. Paraskevas et al. highlighted that the antimicrobial effectiveness of combination mouthwashes relies heavily on achieving a delicate balance between the stability of active ingredients and target specificity [21]. This concept is supported by the observation that solution H, with moderate levels of both CHX and CDO, consistently yielded better MIC results than more concentrated or imbalanced formulations.

The time-kill assay results further supported the MIC and diffusion findings. The mouthwashes G and H sustained bacterial inhibition for up to 24 h across all tested strains. Significant reductions in CFUs were observed at 6 and 24 h (*p* < 0.01), with solutions G and H exhibiting the most sustained effects. In contrast, solutions I and J showed partial regrowth of *Ec* and *Fn* after 6 h, despite their higher CDO content. These findings reinforce the idea that higher concentrations of CDO do not necessarily enhance antimicrobial activity and may compromise formulation stability or specificity.

Additionally, our results suggest that bacterial susceptibility to different CHX-CDO formulations varies by strain. For instance, *Pg* and *Pi* were particularly sensitive to CHX, even at lower concentrations, whereas *Ec* and *Aa* showed greater responsiveness to elevated CDO levels. This variation may reflect differences in cell wall structure, redox sensitivity, or membrane permeability among the species tested. As previous studies have shown, CHX tends to be more effective against Gram-positive bacteria due to the absence of an outer membrane. In contrast, Gram-negative bacteria, such as those tested in this study, often exhibit intrinsic resistance [22–24]. Nonetheless, the efficacy of the CHX-CDO combinations against these anaerobes is promising.

The literature also supports combining CHX with alternative agents to reduce the required CHX concentration. Gränicher et al. and Becker et al. reported that low-dose CHX mouthwash, when enhanced with enzymes or cetylpyridinium chloride (CPC), provided equivalent or superior antibacterial performance compared with CHX 0.2% alone [25, 26]. Likewise, stabilized CDO solutions have been shown to achieve rapid bacterial kill rates while minimizing adverse effects, making them suitable candidates for adjunctive use in oral hygiene products [17, 27].

The promising results of solutions G–J, especially G and H, also support this concept. These mouthwashes maintained high levels of antibacterial efficacy despite containing only 0.01–0.02% CHX. Their performance in both MIC and time-kill assays underscores the potential of using low-CHX, CDO-enriched mouthwashes to manage halitosis while mitigating CHX-associated side effects.

Collectively, the data suggest that the optimal balance of efficacy and safety may be achieved using CHX concentrations of 0.01–0.02% in combination with 0.05% CDO. These ratios appear to maintain antibacterial potency while improving chemical stability and reducing undesirable interactions. However, several limitations must be considered when interpreting these findings. This study was conducted under controlled *in vitro* conditions, which may not fully replicate the complex and dynamic environment of the oral cavity. Only a limited number of representative halitosis-associated strains were tested, and the long-term effects on the oral microbiota or host tissues were not assessed. Furthermore, the chemical stability of CDO in mixed formulations was not quantitatively evaluated.

This research assessed the antibacterial efficacy of the formulations against specific halitosis-related species under regulated *in vitro* conditions. However, tongue coating and dental plaque harbor complex polymicrobial communities, where microbial interactions, metabolic cross-feeding, and structural protection within the biofilm are essential for the synthesis of VSCs [28]. The antimicrobial responses of biofilm-embedded communities are significantly different from those of planktonic monocultures. Recent literature suggests that microbial changes induced by mouthwash should be assessed not only in terms of quantitative reductions in bacteria but also in relation to their impact on the ecological balance of the oral microbiome, as disruptions may result in community shifts or dysbiosis [29]. Moreover, the oral microbiota is increasingly acknowledged as a complex network with potential ramifications for the emergence of antimicrobial resistance [30], underscoring the necessity for strategies that safeguard beneficial taxa.

Consequently, despite the current findings providing significant baseline evidence, they may not fully reflect clinical performance. Future research should use multispecies biofilm models and microbiome sequencing to evaluate the effects of tailored low-dose CHX/0.05% CDO formulations on microbial composition and function in a more physiologically relevant context. Ultimately, clinical validation is necessary to ascertain the enduring effects of these formulations in the management of halitosis.

## Data Availability

The data that support the findings of this study are available from the corresponding author upon reasonable request.

## References

[1] Degif R, Abaynew Y. Knowledge, attitudes, and practices toward halitosis among dental patients at Zewditu Memorial Hospital, Addis Ababa, Ethiopia. Front Oral Health. 2025;6:1522682. 10.3389/froh.2025.1522682 40065837 PMC11891364

[2] Akaji EA, Folaranmi N, Ashiwaju O. Halitosis: a review of the literature on its prevalence, impact and control. Oral Health Prev Dent. 2014;12:297–304. 10.3290/j.ohpd.a33135 25525639

[3] Izidoro C, Botelho J, Machado V, Reis AM, Proença L, Alves RC, et al. Revisiting standard and novel therapeutic approaches in halitosis: a review. Int J Environ Res Public Health. 2022;19:11303. 10.3390/ijerph191811303 36141577 PMC9516975

[4] Kapoor U, Sharma G, Juneja M, Nagpal A. Halitosis: current concepts on etiology, diagnosis and management. Eur J Dent. 2016;10: 292–300. 10.4103/1305-7456.178294 27095913 PMC4813452

[5] Tangerman A, Winkel EG. Intra- and extra-oral halitosis: finding of a new form of extra-oral blood-borne halitosis caused by dimethyl sulphide. J Clin Periodontol. 2007;34(9):748–55. 10.1111/j.1600-051X.2007.01116.x 17716310

[6] Poppolo Deus F, Ouanounou A. Chlorhexidine in dentistry: pharmacology, uses, and adverse effects. Int Dent J. 2022;72:269–77. 10.1016/j.identj.2022.01.005 35287956 PMC9275362

[7] Szalai E, Tajti P, Szabó B, Hegyi P, Czumbel LM, Shojazadeh S, et al. Daily use of chlorine dioxide effectively treats halitosis: a meta-analysis of randomised controlled trials. PLoS One. 2023;18:e0280377. 10.1371/journal.pone.0280377 36634129 PMC9836286

[8] Anna H, Barnabás P, Zsolt L, Romána Z. Tracking of the degradation process of ethylenediaminetetraacetic acid and chlorhexidine digluconate in the presence of hyper-pure chlorine dioxide in endodontic disinfection. J Pharm Biomed Anal. 2019;164:360–4. 10.1016/j.jpba.2018.11.005 30439663

[9] Buyukozer Ozkan H, Terlemez A, Orhan EO. Proton nuclear magnetic resonance spectroscopy analysis of mixtures of chlorhexidine with different oxidizing agents activated by photon-induced photoacoustic streaming for root canal irrigation. Photobiomodul Photomed Laser Surg. 2020;38:374–9. 10.1089/photob.2019.4734 32119810

[10] Thao TTP, Ngan LTM, Thuy PAV. Isolation and identification of porphyromonas gingivalis from sub-gingival plaque samples of patients with periodontitis. Ho Chi Minh City J Med. 2018;22(5):178.

[11] Thao TTP, Ngan LTM, Van NTT, Thuy PAV. Isolation and storage *Aggregatibacter actinomicetemcomitans* from the subgingival plaque of patients with periodontitis. Sci Tech Dev J Health Sci. 2021;2(2):185–93. 10.32508/stdjhs.v2i2.467

[12] Thao TTP, Tham DT, Hung TT. Isolation and storage of *Fusobacterium nucleatum* from the subgingival plaque of patients with periodontitis. HIUJS. 2022;19:31–40.

[13] Brookes ZLS, Bescos R, Belfield LA, Ali K, Roberts A. Current uses of chlorhexidine for management of oral disease: a narrative review. J Dent. 2020;103:103497. 10.1016/j.jdent.2020.103497 33075450 PMC7567658

[14] Mejía K, Rodríguez-Hernández AP, Martínez-Hernández M. Insights into the mechanism of action of chlorhexidine on Porphyromonas gingivalis. Int J Dent. 2025;2025:1492069. 10.1155/ijod/1492069 40223868 PMC11986949

[15] Jefri UHNM, Khan A, Lim YC, Lee KS, Liew KB, Kassab YW, et al. A systematic review on chlorine dioxide as a disinfectant. J Med Life. 2022;15:313–8. 10.25122/jml-2021-0180 35449999 PMC9015185

[16] Deka A, Anil TA, Barua P, Paul R. An ex vivo comparative study determining the bactericidal activity of 3 different irrigants against Enterococcus faecalis. Int J Oral Health Dent. 2017;3:85–8. 10.18231/2395-499X.2017.0020

[17] Noszticzius Z, Wittmann M, Kály-Kullai K, Beregvári Z, Kiss I, Rosivall L, et al. Chlorine dioxide is a size-selective antimicrobial agent. PLoS One. 2013;8:e79157. 10.1371/journal.pone.0079157 24223899 PMC3818415

[18] Ogata N. Denaturation of protein by chlorine dioxide: oxidative modification of tryptophan and tyrosine residues. Biochemistry. 2007;46:4898–911. 10.1021/bi061827u 17397139

[19] Medir M, Giralt F. Stability of chlorine dioxide in aqueous solution. Water Res. 1982;16:1379–82. 10.1902/jop.2008.070630

[20] Andrés CMC, Lastra JMP, Andrés Juan C, Plou FJ, Pérez-Lebeña E. Chlorine dioxide: friend or foe for cell biomolecules? A chemical approach. Int J Mol Sci. 2022;23:15660. 10.1016/0043-1354(82)90221-4 36555303 PMC9779649

[21] Paraskevas S, Rosema NA, Versteeg P, Van der Velden U, Van der Weijden GA. Chlorine dioxide and chlorhexidine mouthrinses compared in a 3-day plaque accumulation model. J Periodontol. 2008;79(8):1395–400. 10.3390/ijms232415660 18672988

[22] Alvarado Rodríguez PY, Rodríguez Zaragoza DE, Ruiz-Reyes H. Comparison of the antimicrobial effect of chlorine dioxide, sodium hypochlorite, and chlorhexidine on bacteria isolated from the root canal. J Dent Oral Sci. 2022;4:1–12. 10.37191/Mapsci-2582-3736-4(4)-141

[23] Nagy E, Boyanova L, Justesen US. How to isolate, identify, and deter- mine antimicrobial susceptibility of anaerobic bacteria in routine laboratories. Clin Microbiol Infect. 2018;24:1139–48. 10.3389/fmicb.2021.741863 29458156

[24] Abbood HM, Hijazi K, Gould IM. Chlorhexidine resistance or cross resistance, that is the question. Antibiotics (Basel). 2023;12:798. 10.3390/antibiotics10060730 37237701 PMC10215778

[25] Gränicher KA, Karygianni L, Attin T, Thurnheer T. Low concentrations of chlorhexidine inhibit the formation and structural integrity of enzyme-treated multispecies oral biofilms. Front Microbiol. 2021;12:741863. 10.1016/j.cmi.2018.02.008 34650542 PMC8506149

[26] Becker K, Brunello G, Scotti L, Drescher D, John G. Efficacy of 0.05% chlorhexidine and 0.05% cetylpyridinium chloride mouthwash to eliminate living bacteria on in situ collected biofilms: an in vitro study. Antibiotics (Basel). 2021;10:730. 10.3390/antibiotics12050798 34204281 PMC8235160

[27] Drake D, Villhauer AL. An in vitro comparative study determining bactericidal activity of stabilized chlorine dioxide and other oral rinses. J Clin Dent. 2011;22:1–5. 21290979

[28] Carda-Diéguez M, Rosier BT, Lloret S, Llena C, Mira A. The tongue biofilm metatranscriptome identifies metabolic pathways associated with the presence or absence of halitosis. NPJ Biofilms Microbiomes. 2022;8(1):100. 10.1038/s41522-022-00364-2 36535943 PMC9763428

[29] Kulis E, Cvitkovic I, Pavlovic N, Kumric M, Rusic D, Bozic J. A comprehensive review of antibiotic resistance in the oral microbiota: mechanisms, drivers, and emerging therapeutic strategies. Antibiotics (Basel). 2025;14:828. 10.3390/antibiotics14080828 40868022 PMC12382797

[30] Brookes Z, Teoh L, Cieplik F, Kumar P. Mouthwash effects on the oral microbiome: are they good, bad, or balanced? Int Dent J. 2023;73(Suppl 2):S74–81. 10.1016/j.identj.2023.08.010 37867065 PMC10690560

